# Supplementary material for: A Universal and Quantitative PCR Strategy for Detection and Epidemiologic Analysis of Canine Papillomavirus (CPV)
Source: Int J Mol Sci. 2025 May 6;26(9):4391. doi: 10.3390/ijms26094391 (PMC12072735; doi:10.3390/ijms26094391)
Supplement: Supplementary file 1 [file ijms-26-04391-s001.zip › ijms-3565429-supplementary.pdf]

Supplementary Table S1: Sequence information related to CPV L1

CPVs	GenBank Accession No	L1 Gene Position in Corresponding CPV Genome	Length of L1 (bp)	The Position of Full-Length or Partial L1 in L1 Gene
CPV1	D55633	6837-8348	1512	1-1512
CPV2	AY722648	6202-7713	1512	1-1512
CPV3	DQ295066	5757-7259	1503	230-917
CPV4	EF584537	5795-7294	1500	230-902
CPV5	FJ492743	6167-7672	1506	230-914
CPV6	FJ492744	6368-7876	1509	233-914
CPV7	FJ492742	6032-7546	1515	227-911
CPV8	NC_016014	5849-7342	1494	224-899
CPV9	JF800656	6051-7556	1506	230-914
CPV10	JF800657	5912-7420	1509	230-914
CPV11	JF800658	5889-7388	1500	233-914
CPV12	JQ754321	5811-7316	1506	233-914
CPV13	JX141478	6249-7763	1515	227-911
CPV14	JQ701802	5978-7474	1497	230-902
CPV15	JX899359	5864-7357	1494	233-914
CPV16	KP099966	5565-7202	1638	362-1043
CPV17	KT272399	6003-7523	1521	227-911
CPV18	KT326919	5896-7398	1503	230-917
CPV19	KX599536	5934-7445	1512	227-911
CPV20	KT901797	5832-7331	1500	230-914
CPV21	MH285952	6185-7705	1521	233-920
CPV22	MH285953	6321-7835	1515	236-917
CPV23	MH285954	6000-7673	1673	395-1076
